# Clinical impact of child life intervention combined with comprehensive nutrition intervention on pain management, nutritional status, and treatment compliance in school-age children with limb fractures

**DOI:** 10.3389/fmed.2025.1658982

**Published:** 2025-12-17

**Authors:** Qin Zhang, Fangqin Jin, Jiangping Weng, Xuting Pan, Qinyan Dong, Yuping Zhao, Jiaojiao Sheng

**Affiliations:** Department of Orthopaedics, The Second Hospital of Jiaxing, Jiaxing, Zhejiang, China

**Keywords:** child life intervention, comprehensive nutrition intervention, limb fractures, nutritional status, pain management

## Abstract

**Background:**

Fractures of the limbs are a common health problem among school-aged children and can cause severe pain as well as emotional issues. Traditional care may not fully meet the comprehensive needs of these children. Therefore, exploring more comprehensive intervention measures is of significant clinical importance.

**Objective:**

This research aimed to evaluate the clinical effect of Child Life intervention combined with a comprehensive nutrition intervention on pain management, nutritional status, and treatment compliance of school-age children with limb fractures.

**Methods:**

A total of 100 school-age children with limb fractures treated in our hospital from March 2024 to September 2024 were selected and randomly divided into a control group (CG) and an observation group (OG) using the random number table method, with 50 cases in each group. The CG received conventional nursing intervention, while the OG received Child Life intervention and comprehensive nutrition intervention on the basis of the CG. The pain scores, serum cortisol levels, treatment compliance, and nutritional status in both groups before and after surgery were compared.

**Results:**

At 12 h and 24 h after surgery, the FLACC scores in the OG were lower than those in the CG (*p* < 0.001, *t* = 5.042; *p* < 0.001, t = 4.836). At 12 h and 24 h after surgery, serum cortisol level in the OG exhibited depletion relative to that in the CG (*p* < 0.001, *t* = 6.049; *p* < 0.001, *t* = 11.662). After intervention, treatment compliance in the OG was higher than that in the CG, indicating statistical significance (*p* = 0.037, *χ*2 = 4.332; *p* = 0.017, *χ*2 = 5.741; *p* = 0.025, *χ*2 = 5.005). At 24 h after surgery, Lc, HGB, PA, and ALB levels in the OG were higher than those in the CG, indicating statistical significance (*p* < 0.01, *t* = 2.717; *p* < 0.001, *t* = 3.433; *p* < 0.001, *t* = 5.023; *p* < 0.001, *t* = 4.230).

**Conclusion:**

A combination of Child Life intervention and comprehensive nutrition intervention can enhance the pain management effect in school-age children with limb fractures, attenuate their pain level, ameliorate their perioperative stress status, and improve their postoperative nutritional status, accelerating their recovery.

## Introduction

Limb fractures are the most common type of unintentional injury in children, with incidence ranging from 2.04 to 11.46% in China (1–3). A substantial number of childhood limb fractures are minor and can be treated with plaster, in severe cases—such as fractures with significant displacement, open fractures, or fractures that have not responded to conservative treatment— surgery becomes the primary treatment method. School-age children’s nervous systems are still developing, and the combination of sustaining a fracture and facing an unfamiliar hospital environment can make them prone to anxiety and fear, which reduces their pain threshold and increases their pain perception (4, 5). According to research reports, the incidence of perioperative negative emotions in school-age children with limb fractures is 50–70%, and more than 60% of children experience significant pain after surgery (6). If appropriate interventions are not implemented, their postoperative recovery may be affected, potentially influencing their learning and daily life. After surgery, children often need to remain in bed for extended periods, and reduced activity can affect the digestive and absorption capacity of the gastrointestinal tract, leading to malnutrition. Once malnutrition occurs, the risk of complications such as infection further affects the recovery (7).

Child Life, also known as pediatric medical counseling, is a developmental intervention based on evidence provided by trained and certified children’s medical counselors for infants, children, adolescents, and families seeking medical treatment. It helps children and their parents cope with stress related to illness and treatment (8). Children are born with the ability to play, and games serve as a language for them. Game-based interventions during the perioperative period can markedly reduce anxiety in hospitalized children, alleviate negative emotions, and mitigate their psychological burden, helping them adapt better to the hospital environment. Such interventions also support their cognitive and emotional development, especially their adaptability, innovation ability, social emotion, and problem-solving abilities (9, 10). The use of childlike toys and role-playing methods can enhance children’s understanding of diagnosis and treatment procedures, alleviate negative emotions, mitigate pain, and improve comfort. Studies have shown that applying game-based interventions to children undergoing chemotherapy yields positive outcomes (11). Thus, Child Life plays a crucial role in pediatric medical auxiliary services. Traditional perioperative nursing does not pay attention to the nutritional status of pediatric patients and provides only basic dietary guidance, which cannot meet actual needs. In contrast, comprehensive nutrition intervention offers more scientific supplementation of nutrients, laying a foundation for improved prognosis (12).

This research aimed to clarify the clinical effect of Child Life intervention combined with comprehensive nutrition intervention on pain management, nutritional status, and treatment compliance of school-age children with limb fractures. The report is as follows.

## Methods

### Study design

This was a prospective randomized controlled study. We used a consecutive sampling method to screen all school-age children with limb fractures who were admitted to our hospital from March to September 2024. After meeting the inclusion criteria and excluding those who met the exclusion criteria, a total of 100 eligible children were selected. These children were then randomly divided into a control group (CG) and an observation group (OG) using the random number table method, with 50 cases in each group.

The inclusion criteria were: (1) age between 6 and 12 years; (2) meeting the diagnostic criteria for limb fractures, including changes in joint shape, bone fricative, abnormal movement at the fracture site, obvious pain and tenderness, and increased pain during movement; (3) presence of fracture lines on X-ray examination; (4) scheduled for surgery under general anesthesia; and (5) parents providing informed consent for participation in the research.

The exclusion criteria included: (1) child patients with multiple fractures; (2) child patients with compound injuries; (3) child patients with pre-existing bone tumors (including both benign and malignant tumors, regardless of whether the fractures are pathological or traumatic); and (4) child patients with cognitive impairment. This research received approval from the Ethics Committee of our hospital.

### Methods

The CG received conventional nursing intervention. Nursing staff should provide conventional oral education to child patients and their parents after admission, including diagnosis and treatment procedures, precautions, disease knowledge, conventional dietary guidance, and functional exercise methods. Nursing staff should visit child patients 1 day before surgery to assess their basic condition, inform them of preoperative water deprivation and fasting time, and prepare the necessary items. Nursing staff should escort child patients to the operating room on the day of surgery. Nursing staff should conduct postoperative evaluations of physical signs and pain level. For child patients with a score of 4 points or higher on the Face Legs Activity Cry Consolability (FLACC) after pediatric surgery, drugs should be applied for pain relief.

The OG received Child Life intervention and comprehensive nutrition intervention basis of CG. (1) Child Life intervention: A. Establish Child Life intervention group. Team members should vote to select a leader for implementing the Child Life intervention, providing training for members, ensuring smooth and effective execution of all intervention measures and avoiding the impact of intervention effects due to staff negligence. B. Develop game intervention plans: Nursing staff should communicate with child patients and their parents to identify the children’s fears. Environmental unfamiliarity, separation anxiety, and fear of postoperative pain are major stressors that lead to fear in child patients. Based on this analysis, nursing staff should design and implement game intervention plans to address these stressors effectively. C. Set up child-friendly game areas: Nursing staff should choose multi-person wards as game areas and decorate them with cartoon stickers, including trees, small animals, and flowers. Moreover, nursing staff should provide children with toys, books, and educational manuals that are suitable for their ages and ensure proper disinfection after use. D. Implement Child Life intervention: The intervention game that was applied before surgery is named “Doctor and Baby.” Game materials that need to be prepared should include dolls, blood pressure monitors, stethoscopes, infusion sets, and adhesive tapes. When playing games, nursing staff should accompany child patients into the wards and perform role-playing; child patients should play the roles of doctors to measure blood pressure for dolls, listen to the heart rate of dolls, and administer intravenous fluids to dolls; a total of approximately 20 min of practice should be conducted to familiarize child patients with medical procedures. The intervention game, which was applied before anesthesia induction, is named “Surgical Game Package”; the game materials that need to be prepared should include educational cards, picture books, infusion sets, oxygen tubes, toy anesthesia beds, dolls, bandages, operating room pictures, and videos. One day before surgery, the nursing staff should display operating room pictures and videos to the child patients and their parents to reduce their unfamiliarity with the operating room environment; nursing staff should guide child patients and their parents through educational cards to abstain from water for 4 h before surgery and from eating for 6 h before surgery. Nursing staff should demonstrate anesthesia, surgical procedures, postoperative dressing changes, oxygen inhalation, and brace fixation with the use of dolls. After the demonstration, they should guide the child patients to retell and deepen them. The intervention game that is applied after completion of surgical treatment is named “Game of Distraction”; game materials that need to be prepared should include blocks, chessboards, watercolor pens, jigsaw puzzles, toy cars, puzzle questions, and plasticine; when child patients return to wards, nursing staff should guide the child patients to take deep breaths and watch their favorite cartoons while playing games; nursing staff should guide family members of child patients to interact with the child patients through provided game materials and provide game guidance for child patients and their parents. A total of approximately 30 min of games should be conducted to distract the children’s attention and alleviate their pain. (2) Comprehensive nutrition intervention: A. Parenteral nutrition: In the early stage of fractures, a semi-liquid diet should be followed, mainly consisting of easily digestible and light foods, and having more meals a day but less food at each, with 4–5 meals a day; recommended diet: steamed eggs + soybean milk for breakfast, noodles with chopped vegetables for lunch, noodle soup, millet congee, and other foods for dinner. In the recovery period of fractures, a high-protein, high-calorie, and nutritious diet should be followed, as well as an increased intake of fat, vitamins, calories, and high-quality protein. B. Enteral nutrition: Nursing staff should calculate the daily energy demand of child patients based on their body weight and age and then develop protein sources and ratios, three major nutrient ratios, and calorie requirements (protein is generally 1.0–1.2 g/kg per day, and calories are generally 25–30 kcal/kg per day). Approximately 24 h after surgery, 20 g of Intact Protein Enteral Nutrition Powder should be dissolved in 100 mL of water and taken orally 5 times a day; nursing staff should monitor the treatment status of child patients. Nursing staff should gradually elevate the dosage of Intact Protein Enteral Nutrition Powder based on nutritional status assessment results and the basic conditions of child patients. If child patients experience symptoms such as indigestion and loss of appetite during enteral nutrition support, nursing staff should adjust the daily dosage and provide targeted treatment. If child patients have difficulty eating, enteral nutrition should be administered through nasogastric tubes.

### Observation indicators

#### Pain scores

The FLACC scores in both groups 1 day before surgery, when returning to the ward after surgery, 12 h after surgery, and 24 h after surgery were compared. The evaluation included five items, namely facial expressions, body movements, leg movements, crying and screaming, and consolability, with a total of 0–10 points. The higher the score, the more severe the pain (13).

#### Serum cortisol level

The serum cortisol concentration in both groups 1 day before surgery, 12 h after surgery, and 24 h after surgery was compared. Approximately 5 mL of fasting venous blood was collected, followed by centrifugation in a JIDI-20D centrifuge at 3000 r/min for 10 min and detection with the chemiluminescence method.

#### Treatment compliance

The compliance of pediatric patients was evaluated with a self-designed orthopedic pediatric treatment compliance scale. The content of the scale includes infusion therapy compliance, functional exercise compliance, dietary compliance, and standardized medication compliance.

#### Nutritional status

The nutritional status in both groups, 1 day before surgery and 24 h after surgery, was compared. Approximately 2 mL of elbow vein blood was collected on an empty stomach in the morning, and the serum was separated, followed by indicator testing through a fully automatic biochemical device. Nutritional indicators include lymphocyte count (Lc), hemoglobin (HGB), prealbumin (PA), and albumin (ALB).

### Statistical analysis

SPSS 27.0 statistical software was used for data analysis. Quantitative data that conform to a normal distribution received representation as (x ± s), followed by a t-test for comparison between groups. Counting data received representation by n (%), followed by the χ2 test for comparison between groups. A *p*-value of < 0.05 indicated a statistically significant difference.

## Results

### Comparison of general data between the groups

The CG consisted of 28 male and 22 female children, and the mean age was 8.25 ± 1.30 years old. There were 20 cases of diaphyseal fracture, 16 cases of supracondylar fracture of the humerus, and 14 cases of humeral lateral condyle fracture. The time from injury to admission was (30.25 ± 3.15) min. Twenty cases underwent open reduction surgery, while 30 cases underwent closed reduction surgery. The OG included 31 male and 19 female children, and the mean age was 8.18 ± 1.25 years old. There were 18 cases of diaphyseal fracture, 18 cases of supracondylar fracture of the humerus, and 14 cases of humeral lateral condyle fracture. The time from injury to admission was (31.06 ± 3.24) min. Eighteen cases underwent open reduction surgery, while 32 cases underwent closed reduction surgery. There was no statistical significance in the general data between the two groups (*p* = 0.388, *χ*2 = 0.744; *p* = 0.934, *t* = 0.083; *p* = 0.894, *χ*2 = 0.222; *p* = 0.208, *t* = 1.267; *p* = 0.680, *χ*2 = 0.169; Table 1), indicating comparability.

**Table 1:** General data in both groups.

Item	CG (*n* = 50)	OG (*n* = 50)	*χ*2/*t*	p
Sex [*n* (%)]			0.744	0.388
Male	28 (56.0)	31 (62.0)		
Female	22 (44.0)	19 (38.0)		
Age (years)	8.25 ± 1.30	8.21 ± 1.25	0.083	0.934
Fracture type [*n* (%)]			0.222	0.894
Diaphyseal fracture	20 (40.00)	18 (36.00)		
Supracondylar fracture of humerus	16 (32.00)	18 (36.00)		
Humeral lateral condyle fracture	14 (28.00)	14 (28.00)		
Time from injury to admission (min)	30.25 ± 3.15	31.06 ± 3.24	1.267	0.208
Operation method [*n* (%)]			0.169	0.680
Open reduction	20 (40.00)	18 (36.00)		
Closed reduction	30 (60.00)	32 (64.00)		

### Comparison of FLACC scores between both groups at different time points

**Figure 1:**
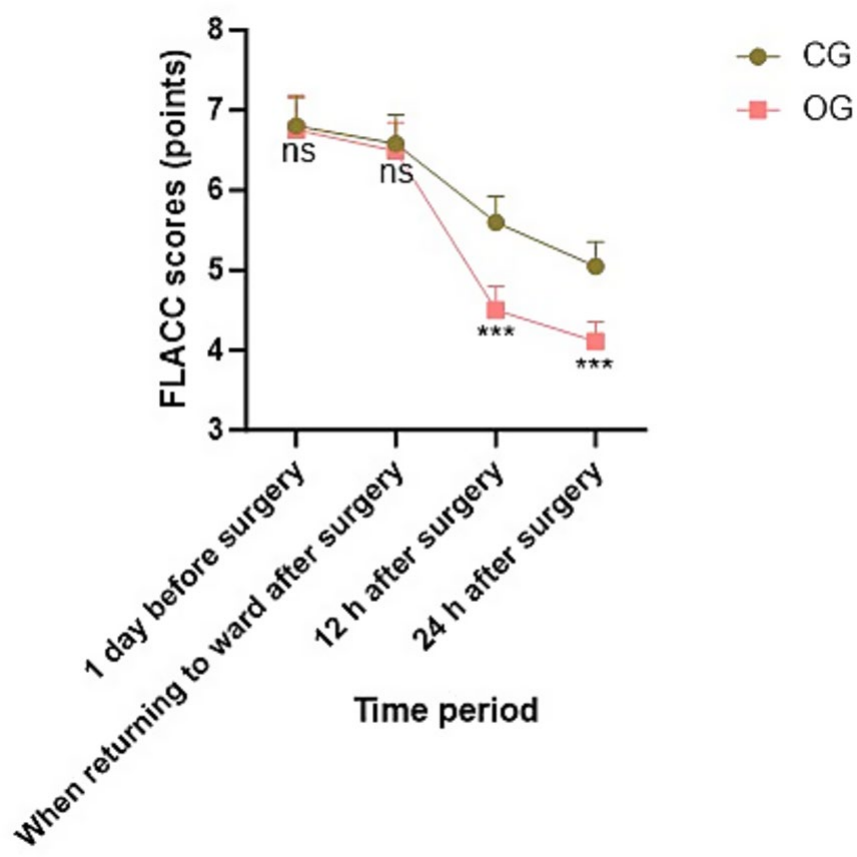
FLACC scores in both groups at different time points. ns meant no significant difference; ***p < 0.001.

No statistical significance in FLACC scores was exhibited between both groups 1 day before surgery and when returning to the ward after surgery (*p* = 0.856, *t* = 0.181; *p* = 0.722, *t* = 0.355). At 12 h and 24 h after surgery, FLACC scores in OG were lower than those in CG, indicating statistical significance (*p* < 0.001, *t* = 5.042; *p* < 0.001, *t* = 4.836; Figure 1).

### Comparison of serum cortisol levels between the groups before and after treatment

**Figure 2:**
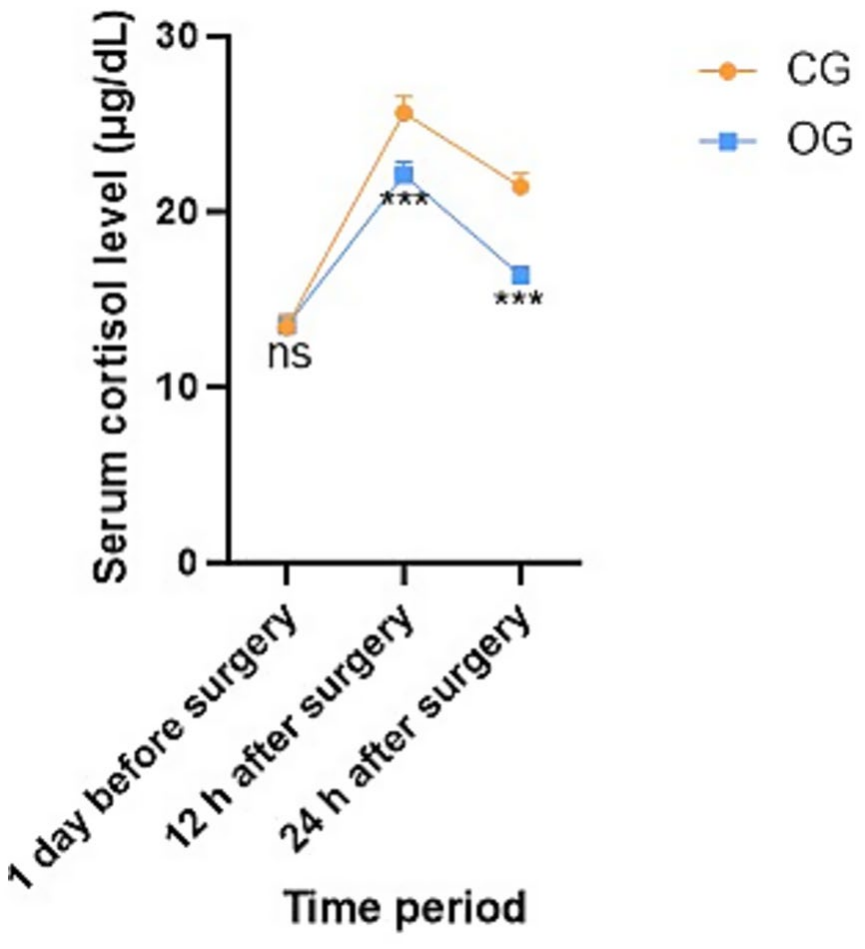
Serum cortisol level in both groups before and after treatment. ns meant no significant difference; ***p < 0.001.

No statistical significance in serum cortisol level was exhibited between both groups 1 day before surgery (*p* = 0.906, *t* = 0.117). At 12 h and 24 h after surgery, serum cortisol level in OG exhibited depletion relative to that in CG, indicating statistical significance (*p* < 0.001, *t* = 6.049; *p* < 0.001, *t* = 11.662; Figure 2).

### Comparison of treatment compliance between the groups

After intervention, the treatment compliance in the OG was higher than that in the CG, indicating statistical significance (*p* = 0.037, *χ*2 = 4.332; *p* = 0.017, *χ*2 = 5.741; *p* = 0.025, *χ*2 = 5.005; Table 2).

**Table 2:** Treatment compliance in both groups.

Groups	*N*	Intravenous therapy compliance [*n* (%)]	Functional exercise compliance [*n* (%)]	Dietary compliance [*n* (%)]	Standardized medication compliance [*n* (%)]
CG	50	40 (80.0)	37 (74.0)	41 (82.0)	38 (76.0)
OG	50	47 (94.0)	46 (92.0)	48 (96.0)	46 (92.0)
*χ*2		4.332	5.741	5.005	4.762
p		0.037	0.017	0.025	0.029

### Comparison of nutritional indicators between the groups before and after treatment

**Figure 3:**
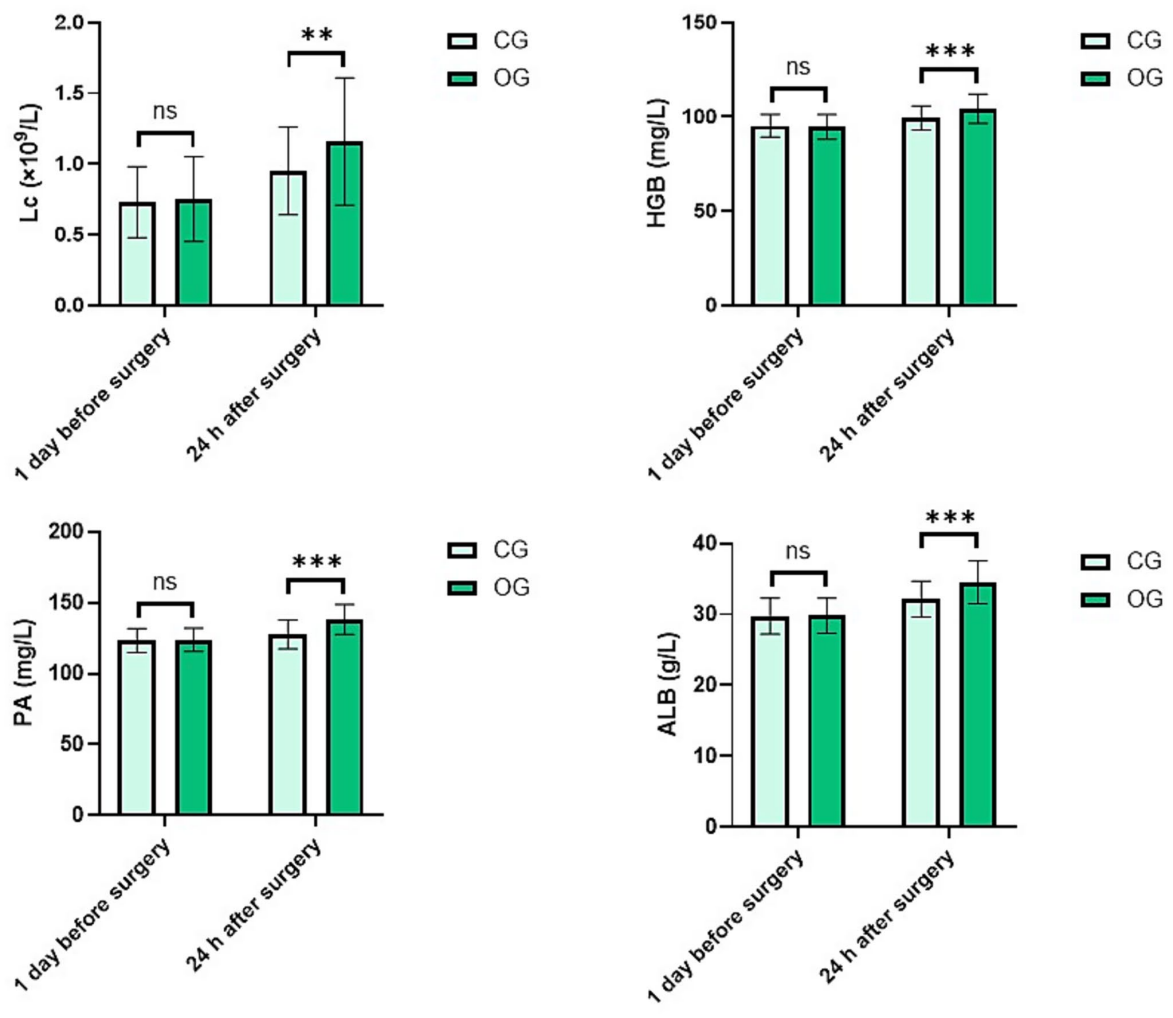
Nutritional indicators in both groups before and after treatment. ns meant no significant difference; **p < 0.01; ***p < 0.001.

No statistical significance in Lc, HGB, PA, and ALB levels was exhibited between both groups 1 day before surgery (*p* = 0.718; *t* = 0.362; *p* = 0.667, *t* = 0.431; *p* = 0.825, *t* = 0.220; *p* = 0.873, *t* = 0.160). At 24 h after surgery, Lc, HGB, PA, and ALB levels in OG were higher than those in CG, indicating statistical significance (*p* < 0.01, *t* = 2.717; *p* < 0.001, *t* = 3.433; *p* < 0.001, *t* = 5.023; *p* < 0.001, *t* = 4.230; Figure 3).

## Discussion

This study aimed to clarify the clinical effect of Child Life intervention combined with a comprehensive nutrition intervention on pain management, nutritional status, and treatment compliance of school-age children with limb fractures. The incidence of limb fractures is high among school-age children (14). The occurrence of limb fractures can result in damage to the bone joint structure of child patients, limiting their mobility. Additionally, surgery inevitably leads to treatment trauma, which can lead to obvious pain in child patients after receiving surgical treatment, especially within 24 h after surgery, when the influence of anesthesia disappears, and pain is most severe.

The appearance of pain can activate the release of cortisol by the hypothalamus–pituitary–adrenal (HPA) axis in the body. As stress level elevates, the concentration of serum cortisol will gradually increase, which can effectively reflect the degree of systemic pain in child patients. When intervening in pain, there are differences in analgesic mechanisms between non-pharmacological interventions and pharmacological interventions. Pharmacological analgesia majorly blocks the pain transmission fiber pathway to achieve pain relief, while game intervention regulates high-level centers of emotions, focus, and memory in child patients to modulate HPA, enhancing the active intensity of the parasympathetic nervous system, reducing the excitability of the sympathetic nervous system, facilitating the secretion of endorphins and dopamine in the body of child patients, and elevating the pain threshold of child patients (15).

In this study, at 12 h and 24 h after surgery, the serum cortisol level in the OG was lower than that in the CG, indicating that Child Life intervention can attenuate serum cortisol levels in school-age children with limb fractures. Moreover, at 12 h and 24 h after surgery, the FLACC scores in the OG were lower than those in the CG, indicating that Child Life intervention can enhance the pain management effect of school-age children with limb fractures and effectively downregulate their pain level. Previous studies have shown a significant correlation between perioperative anxiety and pain levels (16), and that aggravation of anxiety can deepen children’s perception of pain and lower their pain threshold (17). Child Life intervention can facilitate the reduction of pain in child patients through alleviating their anxiety. During Child Life interventions, child patients’ attention receives diversion during the process of playing games, thereby relieving their pain (18). Our study not only confirms these existing findings but also provides empirical data from a specific clinical setting, strengthening the evidence base for the effectiveness of Child Life intervention in pain management. This finding was consistent with previous research by Martos et al., which also demonstrated that a game-based intervention significantly improves preoperative pain in pediatric patients (19). However, our study further specified the time points (12 h and 24 h post-surgery) and the specific patient group (school-age children with limb fractures), providing more detailed and targeted evidence.

The establishment of the Child Life medical model in this research enhanced the treatment compliance of child patients with intravenous infusion, functional exercise, diet, and standardized medication. All games in Child Life involve both child patients and their parents, and nursing staff share strategies to soothe child patients and alleviate their anxiety, thereby enhancing their treatment compliance. This was in line with the study by Yildirim et al. (20), who found that involving parents in pediatric care interventions can improve treatment adherence. However, our study goes a step further by integrating a comprehensive set of treatment aspects (intravenous infusion, functional exercise, diet, and medication) and demonstrating the positive impact of the Child Life model on all these areas simultaneously.

Surgery can cause trauma, elevate the stress response of child patients, and put the body in a high breakdown state, thereby affecting normal glycometabolism and leading to nutritional problems, which is not conducive to postoperative recovery (21). Thus, school-age children with limb fractures not only need to receive surgery and medical game counseling but also comprehensive nutrition intervention to improve their nutritional status, laying the foundation for optimizing prognosis.

Currently, both parenteral nutrition and enteral nutrition can supplement nutrients needed by the body; enteral nutrition has a closer relation to the physiological characteristics of the human body, which can stimulate the release of gastrointestinal hormones and restore gastrointestinal structure and function faster (22, 23). In this study, postoperative comprehensive nutrition intervention (parenteral nutrition + enteral nutrition) was used. Twenty-four hours after surgery, the levels of Lc, HGB, PA, and ALB in the OG were higher than those in the CG, indicating that based on an easily digestible and light semi-liquid diet with parenteral nutrition, enteral nutrition can meet the daily nutritional needs of child patients, better improve their nutritional status, and enable them to recover their appetite and gastrointestinal function as soon as possible. Compared with previous studies that mainly focused on either parenteral or enteral nutrition alone (24, 25), our research demonstrates the synergistic effect of combining both methods in improving the nutritional status of school-age children with limb fractures. This combined approach provides a more comprehensive and effective nutritional support strategy, which is a novel contribution to the field of pediatric postoperative nutrition.

### Limitations and suggestions for future studies

Our study has some limitations. First, the study was carried out in a single hospital, and the sample size, although sufficient for this initial exploration, may not fully represent the broader population of school-age children with limb fractures across different regions, ethnic groups, and socio-economic backgrounds. This could limit the generalizability of the findings. Therefore, we should conduct multicenter studies with larger and more diverse samples to enhance the representativeness and generalizability of the findings. This would allow for a more comprehensive understanding of the effects of combined interventions across different populations.

Second, the assessment of the interventions’ effects was mainly focused on the short-term postoperative period. Long-term effects on pain management, nutritional status, and treatment compliance over weeks or months were not evaluated. Thus, it is unclear whether the observed benefits will be sustained in the long run. Therefore, we should implement long-term follow-up studies to evaluate the sustained effects of Child Life intervention and comprehensive nutrition intervention on school-age children with limb fractures. This would provide valuable information for developing long-term care plans and optimizing treatment strategies.

Third, there may be unmeasured confounding factors that could have influenced the results. For example, the children’s pre-existing health conditions, family support systems, and environmental factors during the recovery period were not comprehensively accounted for, which might have introduced bias into the study. In future studies, we should make a more thorough effort to identify and control for potential confounding factors. This could involve collecting detailed information on the children’s pre-existing health status, family environment, and social support, and using appropriate statistical methods to adjust for these factors during data analysis.

In addition, some of the assessment tools, such as the FLACC score for pain and the evaluation of treatment compliance, have a certain degree of subjectivity. This could lead to measurement errors and affect the accuracy of the results. Therefore, we should explore and incorporate more objective assessment methods for pain, nutritional status, and treatment compliance.

## Conclusion

A combination of Child Life intervention and comprehensive nutrition intervention can enhance the pain management of school-age children with limb fractures, reduce their pain, alleviate perioperative stress, enhance treatment compliance, and improve postoperative nutritional status, thereby accelerating recovery. This approach is worthy of further promotion in clinical practice.

## Data Availability

The original contributions presented in the study are included in the article/supplementary material, further inquiries can be directed to the corresponding author.

## References

[1] Hu D Xu Z Shi T Zhong H Xie Y Chen J. Elastic stable intramedullary nail fixation versus submuscular plate fixation of pediatric femur shaft fractures in school age patients: a PRISMA-compliant systematic review and meta-analysis. Medicine. (2023) 102:e35287. doi: 10.1097/MD.0000000000035287, 37773849 PMC10545301

[2] Ouillette R Bastrom T Newton P Pennock A. Elastic intramedullary nails in the treatment of pediatric length unstable femur fractures. J Pediatr Orthop. (2022) 42:201–8. doi: 10.1097/BPO.0000000000002055, 35089881

[3] Rong C. Clinical progress in the treatment of femoral shaft fracture in children. Adv Clin Med. (2023) 13: 9969–9974. doi: 10.12677/ACM.2023.1361393

[4] Catena N Gennaro GLD Jester A Martínez-Alvarez S Pontén E Soldado F. Current concepts in diagnosis and management of common upper limb nerve injuries in children. J Child Orthop. (2021) 15:89–96. doi: 10.1302/1863-2548.15.200203, 34040654 PMC8138792

[5] Bushnell G Gerhard T Crystal S Olfson M. Benzodiazepine treatment and fracture risk in young persons with anxiety disorders. Pediatrics. (2020) 146:e20193478. doi: 10.1542/peds.2019-3478, 32499386 PMC7329250

[6] Benes G Schmerler J Harris A Margalit A Lee R. Flexible nailing: pushing the indications for diametaphyseal lower-extremity fractures. Medicine. (2024) 103:e37417. doi: 10.1097/MD.0000000000037417, 38489726 PMC10939545

[7] Martins DS Piper HG. Nutrition considerations in pediatric surgical patients. Nutr Clin Pract. (2022) 37:510–20. doi: 10.1002/ncp.10855, 35502496

[8] Wong R Tung K Ho F Wong W Chow C Chan K . Effect of a mobile game-based intervention to enhance child safety: randomized controlled trial. J Med Internet Res. (2024) 26:e51908. doi: 10.2196/51908, 38354042 PMC10902767

[9] Dag N Turkkan E Kacar A Dag H. Children's only profession: playing with toys. North Clin Istanb. (2021) 8:414–20. doi: 10.14744/nci.2020.48243, 34585080 PMC8430366

[10] Rashid A Cheong A Hisham R Shamsuddin N Roslan D. Effectiveness of pretend medical play in improving children's health outcomes and well-being: a systematic review. BMJ Open. (2021) 11:e041506. doi: 10.1136/bmjopen-2020-041506, 33472781 PMC7818823

[11] Masoud A Shaheen A Algabbani M AlEisa E AlKofide A. Effectiveness of exergaming in reducing cancer-related fatigue among children with acute lymphoblastic leukemia: a randomized controlled trial. Ann Med. (2023) 55:2224048. doi: 10.1080/07853890.2023.2224048, 37318119 PMC10274562

[12] Ganmaa D Khudyakov P Buyanjargal U Tserenkhuu E Erdenenbaatar S Achtai C . Vitamin D supplements for fracture prevention in schoolchildren in Mongolia: analysis of secondary outcomes from a multicentre, double-blind, randomised, placebo-controlled trial. Lancet Diabetes Endocrinol. (2024) 12:29–38. doi: 10.1016/S2213-8587(23)00317-0, 38048799

[13] Özen V Şahin A Ayyildiz E Açık M Eyileten T Özen N. Comparison of caudal block and sacral erector spina block for postoperative analgesia following pediatric circumcision: a double-blind, randomized controlled trial. Urol Int. (2024) 108:292–7. doi: 10.1159/000538323 38493772

[14] Qiu X Deng H Zhao Z Zeng S Zeng Y Wang X . Upper limb pediatric fractures in 22 tertiary children's hospitals, China: a multicenter epidemiological investigation and economic factor analysis of 32,832 hospitalized children. J Orthop Surg Res. (2022) 17:300. doi: 10.1186/s13018-022-03159-5, 35658921 PMC9166285

[15] Adams I Jayaweera R Lewis J Badawi N Abdel-Latif M Paget S. Postoperative pain and pain management following selective dorsal rhizotomy. BMJ Paediatr Open. (2024) 8:e002381. doi: 10.1136/bmjpo-2023-002381, 38490692 PMC10946356

[16] Aykut A Salman N Demir Z Eser A Özgök A Günaydın S. The influence of pre-operative pain and anxiety on acute postoperative pain in cardiac surgery patients undergoing enhanced recovery after surgery. Turk J Anaesthesiol Reanim. (2023) 51:491–5. doi: 10.4274/TJAR.2023.231477, 38149367 PMC10758670

[17] Chen J Zhang Y Barandouzi Z Xu W Feng B Chon K . Somatosensory profiles differentiate pain and psychophysiological symptoms among young adults with irritable bowel syndrome: a cluster analysis. Clin J Pain. (2022) 38:492–501. doi: 10.1097/AJP.0000000000001046, 35686579 PMC9205184

[18] Hashmi S Paine A Hay D. Seven-year-olds' references to internal states when playing with toy figures and a video game. Infant Child Dev. (2021) 30:e2223. doi: 10.1002/icd.2223, 34483746 PMC8404204

[19] Suleiman-Martos N García-Lara RA Membrive-Jiménez MJ Pradas-Hernández L Romero-Béjar JL Dominguez-Vías G . Effect of a game-based intervention on preoperative pain and anxiety in children: a systematic review and meta-analysis. J Clin Nurs. (2022) 31:3350–67. doi: 10.1111/jocn.16227, 35075716 PMC9787560

[20] Yildirim M Koroglu E Yucel C Kirlak S Sen S. The effect of hospital clown nurse on children's compliance to burn dressing change. Burns. (2019) 45:190–8. doi: 10.1016/j.burns.2018.08.033, 30249435

[21] Wechselberger S Compton F Schilling J. Impact of continuous veno-venous hemoDIALysis with regional citrate anticoagulation on non-NUTRItional calorie balance in patients on the ICU-the NUTRI-DAY study. Nutrients. (2022) 15:63. doi: 10.3390/nu15010063, 36615721 PMC9824471

[22] Brown T Johnson T Gomes A Samavat H Byham-Gray L. Knowledge and clinical practice of ASPEN registered dietitian nutritionist members regarding blenderized tube feedings. Nutr Clin Pract. (2024) 39:651–64. doi: 10.1002/ncp.11145, 38506319

[23] Jimenez E Lamers-Johnson E Long J McCabe G Ma X Woodcock L . Predictive validity of the academy of nutrition and dietetics/American society for parenteral and enteral nutrition indicators to diagnose malnutrition tool in hospitalized adults: a cohort study. Am J Clin Nutr. (2024) 119:779–87. doi: 10.1016/j.ajcnut.2023.12.012, 38432715

[24] Xu B Chen H Zhang Q Chen P. Supplemental parenteral nutrition improves patient outcomes after esophageal cancer surgery: a single-center randomized controlled study. Medicine (Baltimore). (2022) 101:e31893. doi: 10.1097/MD.0000000000031893, 36451459 PMC9704877

[25] Wang J Yu K Zeng Y. Early enteral nutrition intervention promotes multiple functional recovery in patients with traumatic intracerebral hemorrhage: a prospective randomized controlled study. Clin Neurol Neurosurg. (2023) 234:108010. doi: 10.1016/j.clineuro.2023.108010, 37857236

